# Divergent molecular and growth responses of young “Cabernet Sauvignon” (*Vitis vinifera*) plants to simple and mixed infections with *Grapevine rupestris stem pitting-associated virus*

**DOI:** 10.1038/s41438-019-0224-5

**Published:** 2020-01-01

**Authors:** M. Tobar, N. Fiore, A. G. Pérez-Donoso, R. León, I. M. Rosales, M. Gambardella

**Affiliations:** 1 Pontificia Universidad Católica de Chile, Facultad de Agronomía e Ingeniería Forestal, Vicuña Mackena 4860, Macul, Santiago, 7820436 Chile; 2 Universidad de Chile, Facultad de Ciencias Agronómicas, Avenida Santa Rosa 11315, La Pintana, Santiago, 8820808 Chile

**Keywords:** Biotic, Plant physiology

## Abstract

*Grapevine rupestris stem pitting associated virus* (GRSPaV) is one of the most widely distributed viruses; even so, little is known about its effect on *Vitis vinifera*. To provide new insights, the effects of single and mixed GRSPaV infections on the *V. vinifera* cultivar “Cabernet Sauvignon” were studied by evaluating growth parameters, such as measurements of the total plant length, the number and distance of internodes and the number of leaves per shoot. In addition, parameters relating to gas exchange, *i.e*., the stomatal conductance, net photosynthetic rate, internal CO_2_ concentration and leaf transpiration, were also assessed. All the measurements were performed in one- and two-year-old plants with a single GRSPaV infection or mixed infections of GRSPaV and *Grapevine fanleaf virus* (GFLV). The results show that the plant phytosanitary status did not significantly alter the growth and gas exchange parameters in one-year-old plants. However, in two-year-old plants, single GRSPaV infections increased shoot elongation, which was accompanied by the overexpression of genes associated with the gibberellic acid response pathway. The gas exchange parameters of these plants were negatively affected, despite exhibiting higher *LHCII* gene expression. Plants with mixed infections did not have modified growth parameters, although they presented a greater reduction in the primary photosynthetic parameters evaluated with no change in *LHCII* expression. The results presented here confirm the co-evolution hypothesis for *V. vinifera* and GRSPaV during the early stages of plant development, and they provide new evidence about the effects of GRSPaV and GFLV co-infections on the “Cabernet Sauvignon” cultivar.

## Introduction

The grapevine (*Vitis vinifera* spp.) is one of the most important crops in the world. Its economic relevance has positioned it as one of the most studied fruit species in agricultural science, which has allowed researchers to identify ~70 different viruses to date that infect this species^1^. The *grapevine rupestris stem pitting associated virus* (GRSPaV) is one of the most ubiquitous and variable viruses, and it is capable of infecting several species in the *Vitis* genus^2,3^. The presence of GRSPaV has been closely related to the development of rupestris stem pitting syndrome, which belongs to the rugose wood grapevine disease complex^4,5^, as well as the “vein necrosis” disease observed under a “Richter-110” indicator^6^, and some other disorders with varying levels of severity^7^. However, sufficient evidence to confirm that this virus is the causal agent of these diseases is still lacking.

In addition, a high percentage of GRSPaV infected plants do not develop visible symptoms. Several publications have studied the effect of this virus on different grapevine cultivar; in general, all these studies reported that the presence of the virus did not have a negative effect on plant growth of “Albano”^8^, “Madeleine Sylvaner”, “Ortega”^9^ and “Savagnin rose”^10^ grapevines.

The effect of GRSPaV infection on the physiological performance of the plant and their impact on productivity parameters was also evaluated. Some studies have shown that the presence of this virus in asymptomatic grapevines would not affect the yield or the chemical characteristics of the grape berry in different evaluated cultivars. In some cases, there were differences depending on factors such as the cultivar and the climatic conditions in which the experiment was performed^9^.

A study performed in 2012 in Italy found no effect from GRSPaV on the yields of “Bosco” grapevines. Additionally, the authors presented a complete analysis of the GRSPaV effect on the physiological parameters of grapevines. The results showed that infected plants had a lower chlorophyll content in the leaves and a reduced net photosynthetic rate (*P*_n_)^11^. In this same study, a transcriptomic analysis of the leaves showed that GRSPaV-infected plants presented a higher basal expression of genes associated with the photosynthetic process, such as the genes encoding *rubisco activase* (*RCA)*, *light-harvesting complex I* (*LHCI)*, and *light-harvesting complex II (LHCII)*, or genes related to carbon fixation such as *glyceraldehyde-3-phosphate dehydrogenase (GAPDH), fructose-bisphosphatase (FBPase)* and *ribulose 1,5 bisphosphate carboxylase (RuBP)*. Another category that displayed an interesting behaviour was the stress response gene group, in which most overexpressed genes belonged to the abiotic stress response group while most of the repressed genes fit in the biotic stress response classification. These results led the authors to hypothesize about the possible beneficial effect of the virus, which would produce a basal over-expression of the response against abiotic factors^11^.

Subsequent studies have addressed the response of GRSPaV-infected plants under water stress, finding that individuals with latent GRSPaV infections have a differential profile in their miRNAs expression that allowed the plants to have a greater resilience to initial levels of water stress. This expression was accompanied by modifications in some eco-physiological parameters such as an increased cell density and stomata number, which would confirm a close plant-virus co-evolution^12^. These results lead the authors to propose a symbiotic mutualistic relationship between GRSPaV and *V. vinifera*, in which the presence of the virus would confer a greater capacity to cope with the initial levels of abiotic stress to the plant, among other traits, due to the basal induction of genes related to the abiotic stress response. This approach would confer an adaptive advantage to the plant; however, the transcriptomic analyses performed by Gambino et al.^11^ also showed the basal repression of a large number of genes associated with the defence response against biotic stresses, an issue that, in our opinion, has not been sufficiently explored and that could modify this hypothesis. Especially because a close and inverse association between plant growth and the activation of defence response has been described^13^.

The consequences of single and mixed GRSPaV infections on plant development and gas exchange parameters of *V. vinifera* cv. “Cabernet Sauvignon”, a widely planted grapevine variety around the world, were studied in this work. The aim of the study was to provide new insights on plant-virus interaction and improving the understanding of the effects of GRSPaV infection on grapevine.

## Results

To evaluate the possible effects of GRSPaV on grapevines with single and mixed infections, measurements of the growth and photosynthetic parameters and the expression levels of the genes involved in these processes were performed.

### Genetic analyses of viral isolates from plants with GRSPaV and GRSPaV+GFLV

**Fig. 1:**
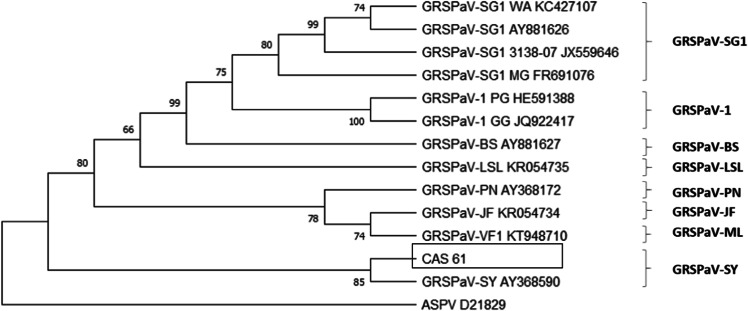
Phylogenetic tree of GRSPaV sequence variants based on partial sequence of the RdRP region of the replicase polyprotein gene. Sequence isolated 61 was obtained from RT-PCR using broad-spectrum primers RSP35 and RSP36. Corresponding sequences from reference isolates were obtained from each of the GenBank complete genome sequences and included in the analysis (Supplementary Fig. 1). The nucleotide sequence corresponding region of Apple stem pitting virus (ASPV, accession number D21829) was retrieved from GenBank and used as an outgroup. Phylogenetic analysis was performed using both the Neighbour Joining (shown here) and the Maximum Likelihood methods. Each cluster is designated in accordance with the nomenclature of GRSPaV variants proposed by Men and Rowhani^14^. Bootstrap values of 50 % or greater (of 1000 replication) are provided.

**Fig. 2:**
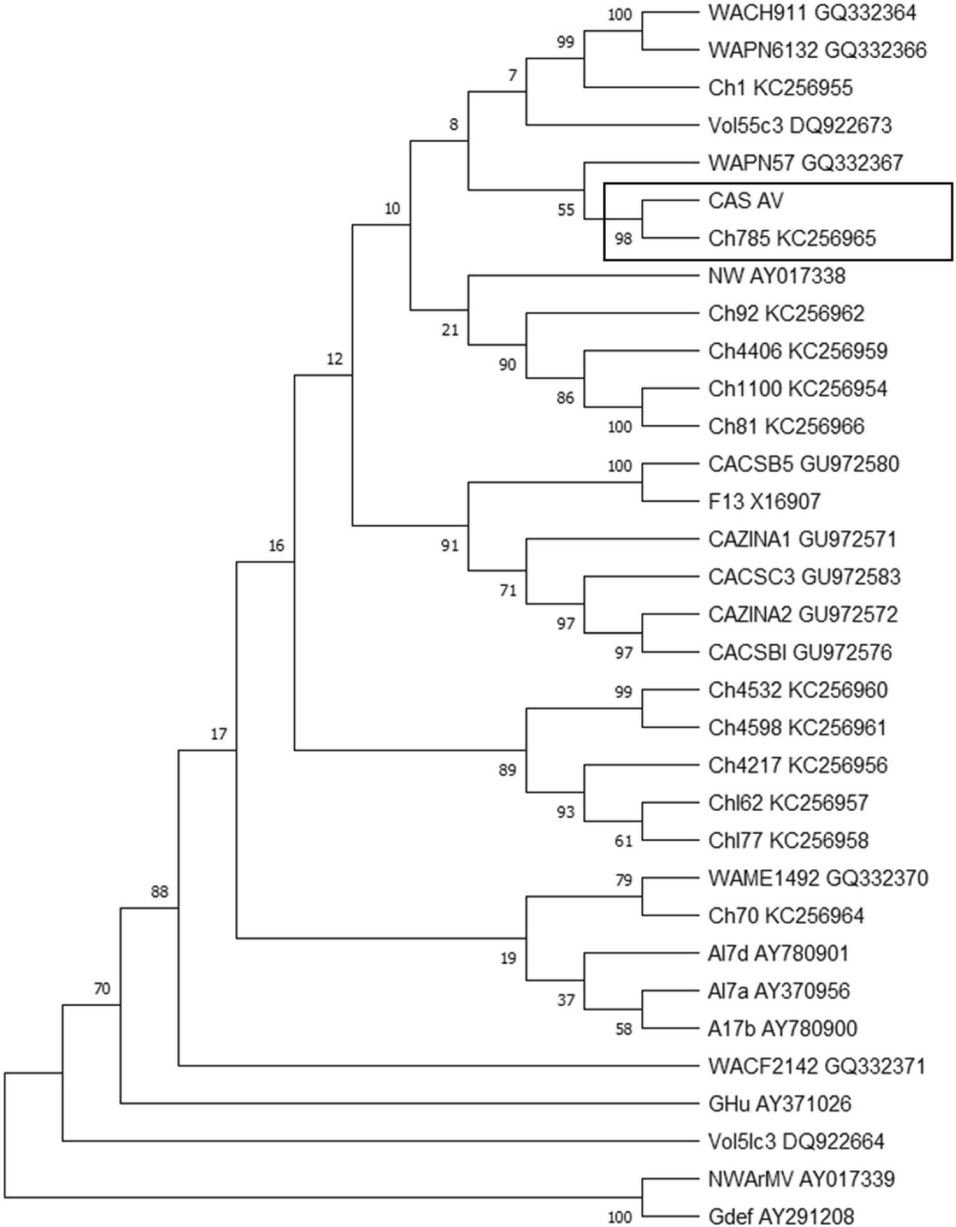
Phylogenetic tree of GFLV sequence variants based on the complete sequence of the coat protein (CP) of the polyprotein gene 2. Sequence isolated CAS AV was obtained from RT-PCR using two pairs of primers CP1 F-CP2 R and CP3 F-CP4R. Twelve entire RNA 2 coding nucleotide sequences of Chilean isolates (Ch, Supplementary Table 2) and corresponding sequences from reference isolates were obtained from each of the GenBank complete genome sequences and included in the analysis (Supplementary Table 3). The nucleotide sequence corresponding region of Arabic mosaic virus (ArMV, accession number AY017339) was retrieved from GenBank and used as an outgroup. Phylogenetic analysis was performed using both the Neighbour Joining (shown here) and the Maximum Likelihood methods. Bootstrap values of 50% or greater (of 500 replication) are provided. Isolate CAS AV corresponded to the Chilean isolates cluster.

To identify the GRSPaV variants present in infected plants, viral RNA was extracted and sequenced. The nucleotide sequence obtained was named “isolate CAS 61” and was compared with sequences present in the GeneBank database. The genetic analysis was performed using the RdRP region of the replicase polyprotein of GRSPaV. The genetic variability analysis indicates that the isolated CAS 61 has a high sequence identity (93%) with the viral strain GRSPaV-SY (Fig. 1). The description of the reference isolates used in the phylogenetic analysis is in Supplementary Table 1. Besides, to characterise the GFLV present in the plant material here used, the genetic material was extracted and sequenced (isolate CAS AV). A phylogenetic analysis was performed based on the complete sequence of the coat protein (CP) of the polyprotein gene 2 (Fig. 2). The results showed a high sequence identity of CAS AV (98%) with another Chilean isolate of GFLV (Ch785). The descriptions of Chilean and reference isolates used in the phylogenetic analysis are in Supplementary Table 2 and 3, respectively.

### Growth monitoring in plants with single and mixed GRSPaV infections

The effect of GRSPaV on plants with single and mixed viral infections was studied by performing growth monitoring on “Cabernet Sauvignon” grapevines with three different phytosanitary statuses: virus-free, GRSPaV-infected and GRSPaV- and GFLV-infected plants.

**Fig. 3:**
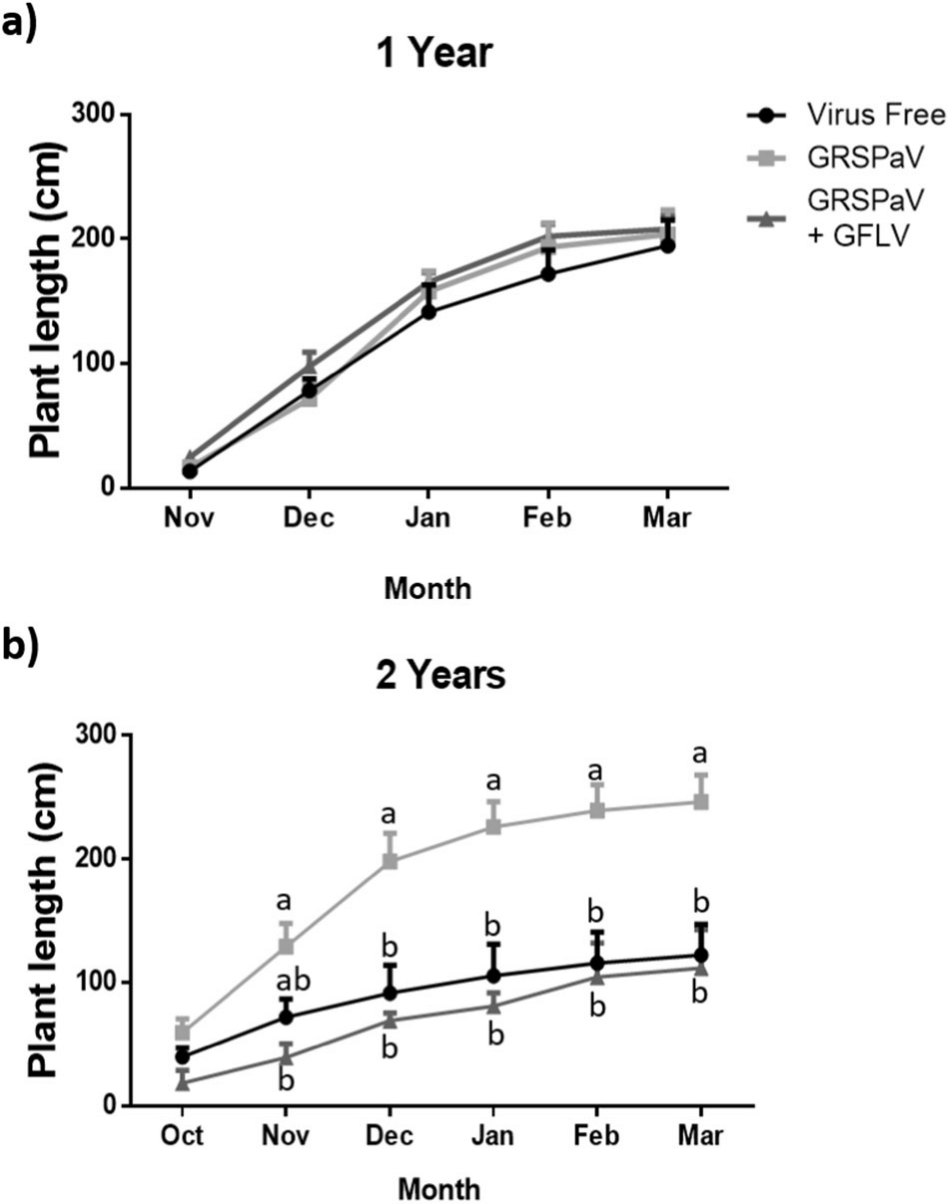
Monthly monitoring of shoot elongation in 1-year and 2-year-old plants. The monthly growth was measured in 1 (**a**) and 2-year-old plants (**b**) with three phytosanitary conditions: virus-free, infected with GRSPaV and infected with GRSPaV and GFLV during the 2017–2018 growth period. The data include three to eight replicates and were analysed by ANOVA, and the graphs show the mean ± SE. Different letters indicate significant differences.

The total shoot length results are shown in Fig. 3. One-year-old plants with different viral infections have similar shoot elongation results relative to virus-free plants throughout the season (Fig. 3a). However, in two-year-old plants, a difference in the shoot elongation was observed since very early in the season; the GRSPaV-infected plants exhibited significantly higher shoot elongation than the control (Fig. 3b). In addition, it is possible to observe that healthy plants and plants with mixed infections displayed the same total shoot elongation, statistically, in both one- and two-year-old grapevines.

**Fig. 4:**
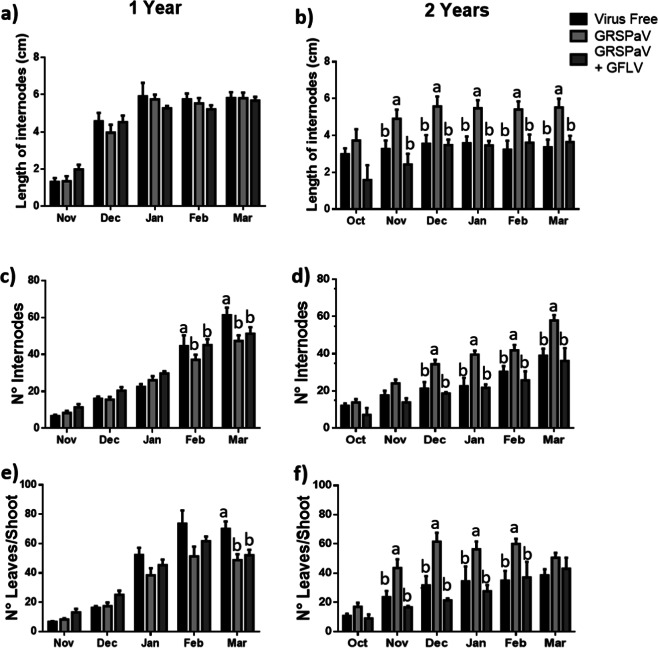
Monthly monitoring of growth parameters in 1-year and 2-year-old plants. The average lengths of the internodes (**a**, **b**), the number of internodes (**c**, **d**) and the number of leaves per shoot (**e**, **f**) were measured in 1 (**a**, **c** and **e**) and 2-year-old plants (**b**, **d** and **f**) under three phytosanitary conditions: virus-free, infected with GRSPaV and infected with GRSPaV and GFLV. The data include three to eight replicates and were analysed by ANOVA, and the graphs show the mean ± SE. Different letters indicate significant differences.

The second growth parameter evaluated here was the average internode length in one- and two-year-old plants. In the first case, no differences were observed in the average lengths of the internodes regardless of the phytosanitary status during the season, except during the first month of evaluations, when significantly higher magnitudes were observed in plants with mixed infections in comparison to the other conditions (Fig. 4a). The results from the two-year-old plants showed that the individuals with simple GRSPaV infections exhibited a significantly longer average internode length than the other conditions evaluated here. This result was consistent throughout the season (Fig. 4b).

When the number of internodes throughout the shoot was observed (Fig. 4 c, d), the results were similar to those obtained for the previously described parameters. In 1-year-old plants, there were no differences in the internode numbers when comparing the different phytosanitary conditions (Fig. 4c). However, in 2-year-old plants, the grapevines infected only with GRSPaV presented a greater number of internodes than the other two groups of evaluated plants (Fig. 4d). In addition, there were no observed differences when comparing the number of internodes in virus-free plants and plants with mixed viral infections.

Regarding the number of leaves per shoot (Fig. 4e, f), the results were similar to the previously described ones, although at the end of the season, a greater number of leaves was observed in virus-free grapevines from one-year-old plants in comparison to the other phytosanitary conditions. In two-year-old plants, the group with a significantly higher number of leaves was the group of plants infected with GRSPaV (Fig. 4f).

To establish any type of correspondence between these results and the viral load, the GRSPaV and GFLV concentrations were quantified in samples collected in both January and March from one-year and two-year-old plants. The results show that there were no statistically significant differences in the viral loads of the samples under any of the conditions or ages under analysis (Supplementary Fig. 1).

### Evaluating gas exchange parameters in plants with single and mixed infections of GRSPaV

**Fig. 5:**
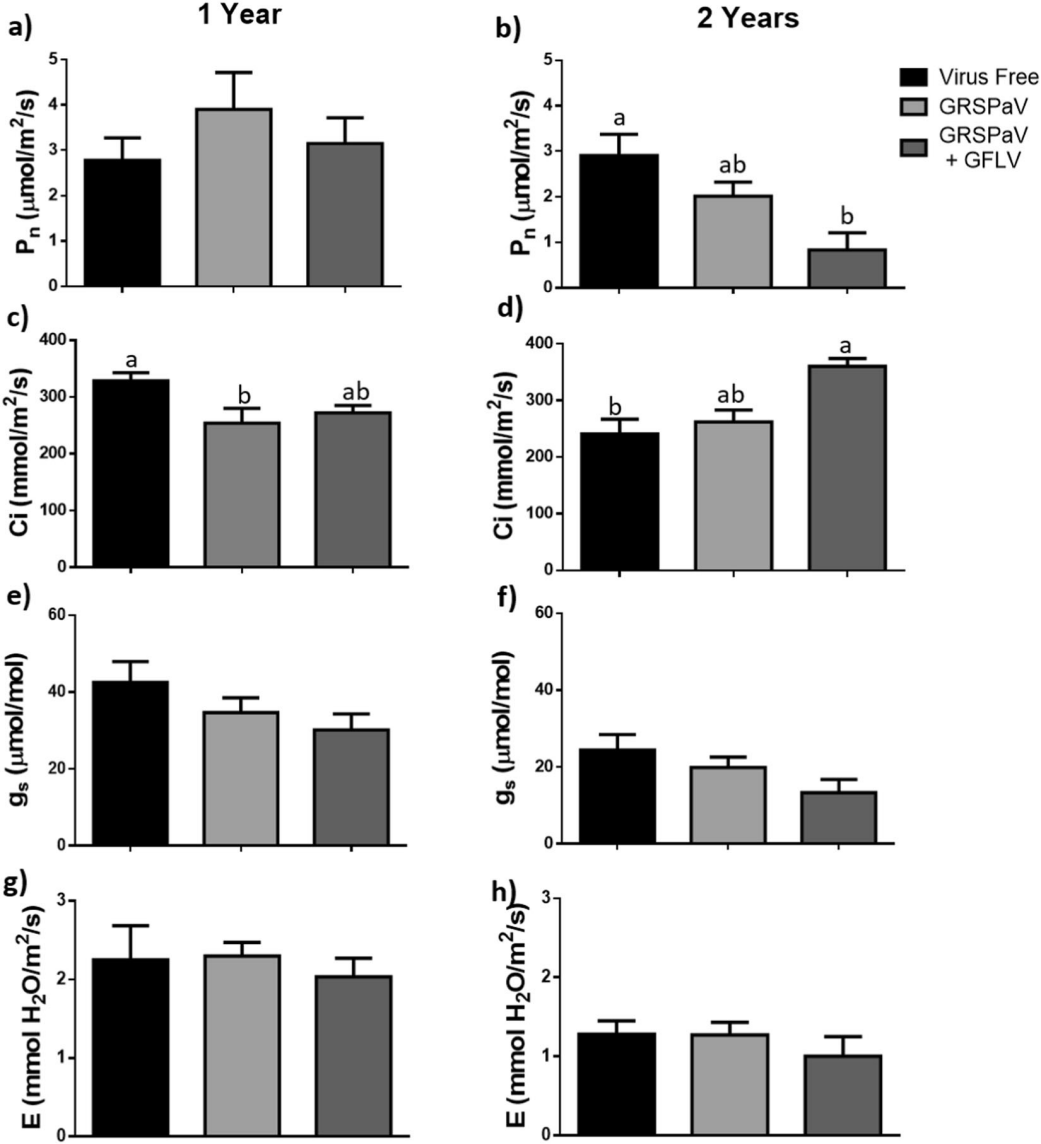
Measurements of gas exchange parameters. The net photosynthesis rate (*P*_n_) (**a**, **b**), internal concentration of leaf CO_2_ (*C*_i_) (**c**, **d**), stomatal conductance (*g*_s_) (**e**, **f**) and transpiration rate of the leaf (*E*) (**g**, **h**) were measured in 1-year-old (**a**, **c**, **e** and **g**) and 2-year-old plants (**b**, **d**, **f** and **h**) with three phytosanitary conditions: virus-free, infected with GRSPaV and infected with GRSPaV and GFLV. The data include three technical repetitions and were analysed by ANOVA and the graphs show the mean ± SE. Different letters indicate significant differences.

Fig. 5 shows the results obtained for the gas exchange parameters evaluated here (the *P*_n_, *C*_i_, *g*_s_ and *E*). The *P*_n_ levels in one-year-old plants exhibited no differences between the three different phytosanitary statuses; however, in two-year-old plants, those with mixed infections yielded significantly lower *P*_n_ values. When the *C*_i_ was measured in one-year-old plants, a significant reduction of this parameter was observed in GRSPaV-infected plants, whereas in two-year-old plants, the group with double viral infections showed a higher *C*_i_ value. The *g*_s_ and *E* results did not show significant differences when comparing the different phytosanitary statuses or plant ages.

### Quantifying levels of gene expression in plants with single and mixed GRSPaV infections

To study if simple and mixed GRSPaV infections can affect the expression of genes involved in metabolic pathways that are directly related to the growth, photosynthetic processes and defence response of the plant, an expression level quantification was performed for selected genes. The gene expression was analysed in samples collected in January (BBCH 75)^15^ and March (BBCH 89)^15^ from 1- and 2-years old plants with three different phytosanitary status.

**Fig. 6:**
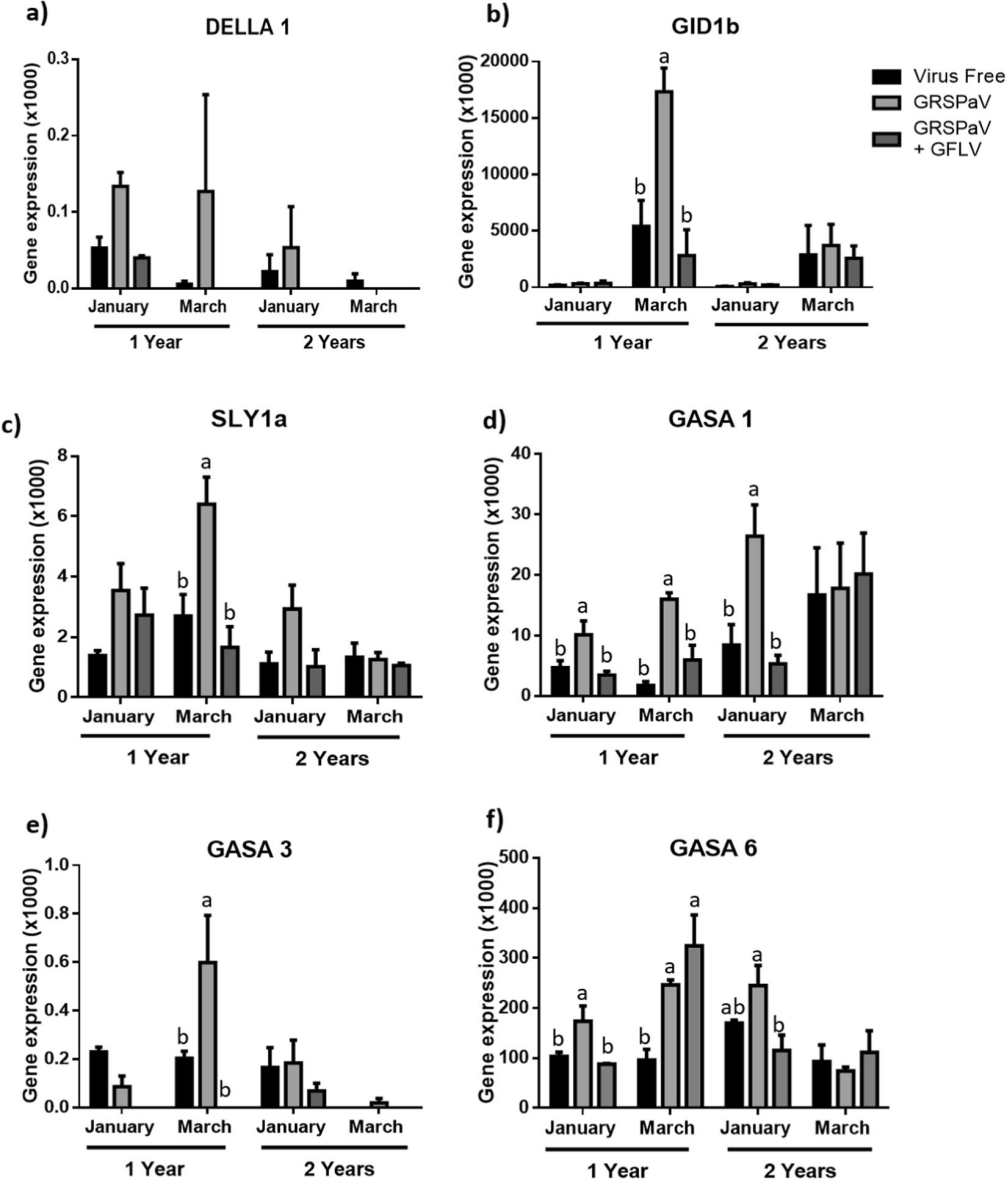
Relative expression of genes associated with the GA response pathway. The relative expression of regulator genes of the GA response, *DELLA1* (**a**), *GID1b* (**b**) and *SLY1a* (**c**), was evaluated. Additionally, the relative expression of genes downstream of the GA stimulus was evaluated for *GASA1* (**d**), *GASA3* (**e**) and *GASA6* (**f**). The graphed expression levels were evaluated in January (BBCH 75) and March (BBCH 89) of 2018 in 1- and 2-years old plants and are presented relative to the reference gene, ubiquitin, and include three biological replicates and two technical repetitions. The data were analysed by one-way ANOVA to compare between phytosanitary conditions. The graphs show the mean ± SE. Different letters indicate significant differences.

In relation to the plant growth, the genes involved in the gibberellic acid (GA) response pathway were selected. Fig. 6 shows the relative expression levels of genes encoding some of the most important regulators of GA pathways, namely *DELLA1, GID1b* and *SLY1a* (Fig. 6a, b and c, respectively). In addition, the expression of the following genes downstream from the GA signal was evaluated: *GASA1, GASA3* and *GASA6* (Fig. 6d, e and f, respectively).

For *DELLA1* (Fig. 6a) the observed differences were not significant for any of the comparisons, and the gene expression levels presented a high standard deviation. The two-way ANOVA also found no differences associated with the age or phytosanitary condition of the plants (Supplementary Table 4). The expression levels of the *GID1b* gene (Fig. 6b) were higher in samples collected in March from one-year-old plants infected with GRSPaV in comparison to the other phytosanitary conditions. This difference was not repeated for the other evaluated time points, although the trend was maintained in March for two-year-old plants, with gene expression levels that were significantly lower than those of the one-year-old plants (Supplementary Table 5). The same behaviour was observed in the expression levels of *SLY1a* (Fig. 6c). Finally, the two-way ANOVA shows that the *SLY1a* expression levels during March in one-year-old plants were significantly higher than the levels quantified in the two-year-old plants (Supplementary Table 6).

In evaluations on the expression of genes downstream from the GA signal, *GASA1* was significantly more highly expressed in GRSPaV-infected plants. This trend was observed during January and March in one-year-old plants and in January in two-year-old plants (Fig. 6d). Additionally, the two-way ANOVA shows that there was a higher transcript level in the two-year-old plants when comparing samples between the differently aged plants collected in January. The same results were obtained when comparing the expression levels of *GASA1* between plants of different ages in March (Supplementary Table 7). For *GASA3*, higher gene expression was observed in one-year-old plants infected with GRSPaV during March (Fig. 6e). Similar *GASA3* expression levels were observed in one- and two-year-old plants during January, whereas in samples collected on March, a significantly higher gene expression was observed in one-year-old plants (Supplementary Table 8). For *GASA6*, the gene was overexpressed in GRSPaV-infected plants at most of the analysed time points except for March, in the two-year-old plants (Fig. 6f). The two-way ANOVA shows that in a comparison of *GASA6* expression levels in samples collected in January, this gene was significantly more highly expressed in two-year-old plants. No differences were found between the samples collected in March from differently aged plants (Supplementary Table 9).

**Fig. 7:**
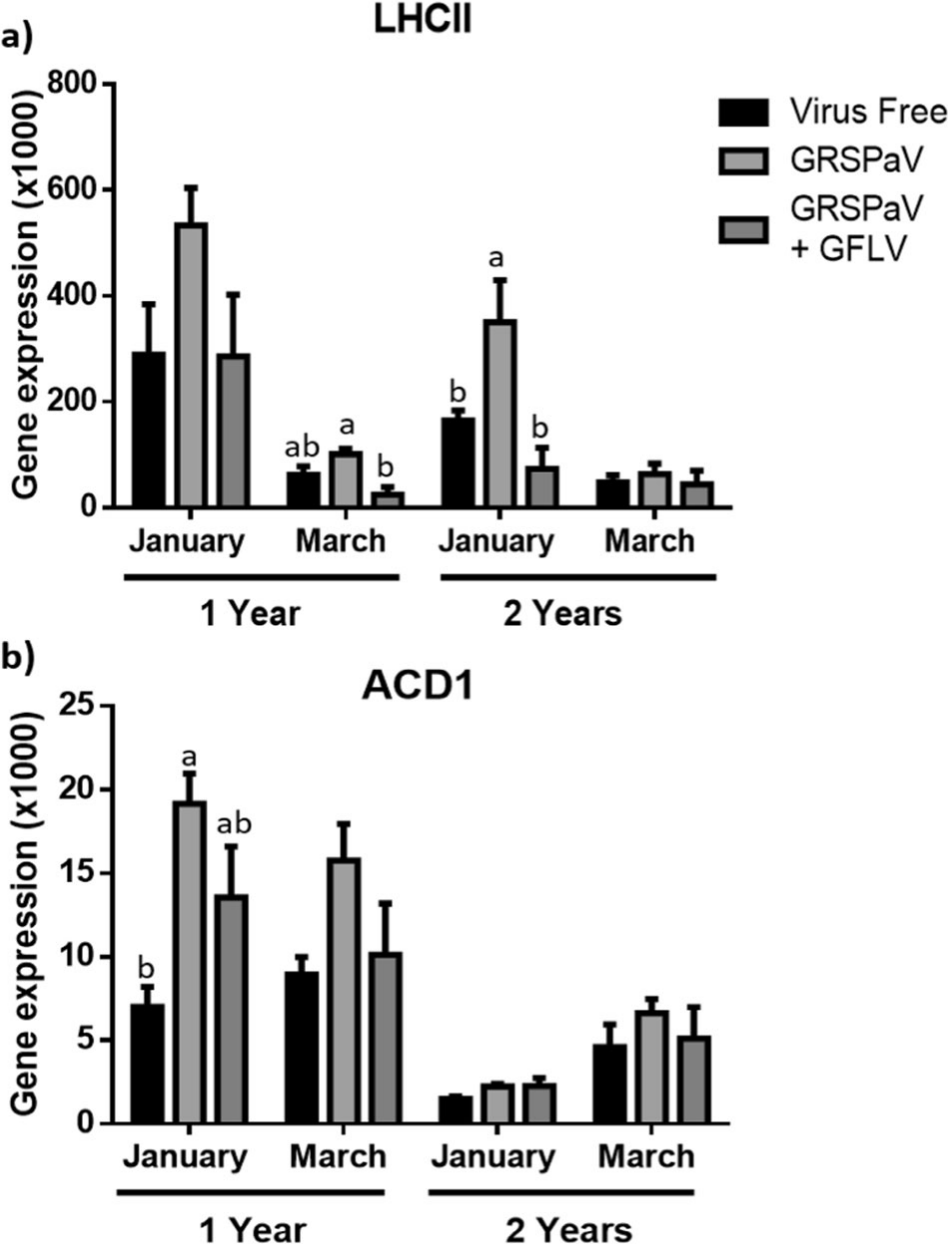
Relative expression of genes involved in photosynthetic processes. The relative expression of *LHCII* (**a**) and *ACD1* (**b**) was evaluated. The graphed expression levels were evaluated in January (BBCH 75) and March (BBCH 89) on 2018 in one- and two-year-old plants and are presented in relation to the reference gene, ubiquitin, and include three biological replicates and two technical repetitions. The data were analysed by one-way ANOVA to compare between phytosanitary conditions. The graphs show the mean ± SE. Different letters indicate significant differences.

The next measurements to be performed were the relative quantifications of genes related to the photosynthetic process (*LHCII*) and chlorophyll degradation (*ACD1*) (Fig. 7). For *LHCII* (Fig. 7a), higher gene expression was observed in GRSPaV-infected plants with respect to the other evaluated conditions, although the most significant difference was only in March for one-year-old plants and in January for two-year-old plants. The two-way ANOVA showed significant differences in the *LHCII* expression between leaves from one- and two-year-old plants collected during January (Supplementary Table 10). For *ACD1* (Fig. 7b), a similar result was observed in one-year-old plants, and there were no differences in *ACD1* expression when comparing different phytosanitary statuses within the two-year-old plant group. Additionally, the two-way ANOVA shows that one-year-old plants have greater levels of *ACD1* expression than two-year-old plants (Supplementary Table 11).

**Fig. 8:**
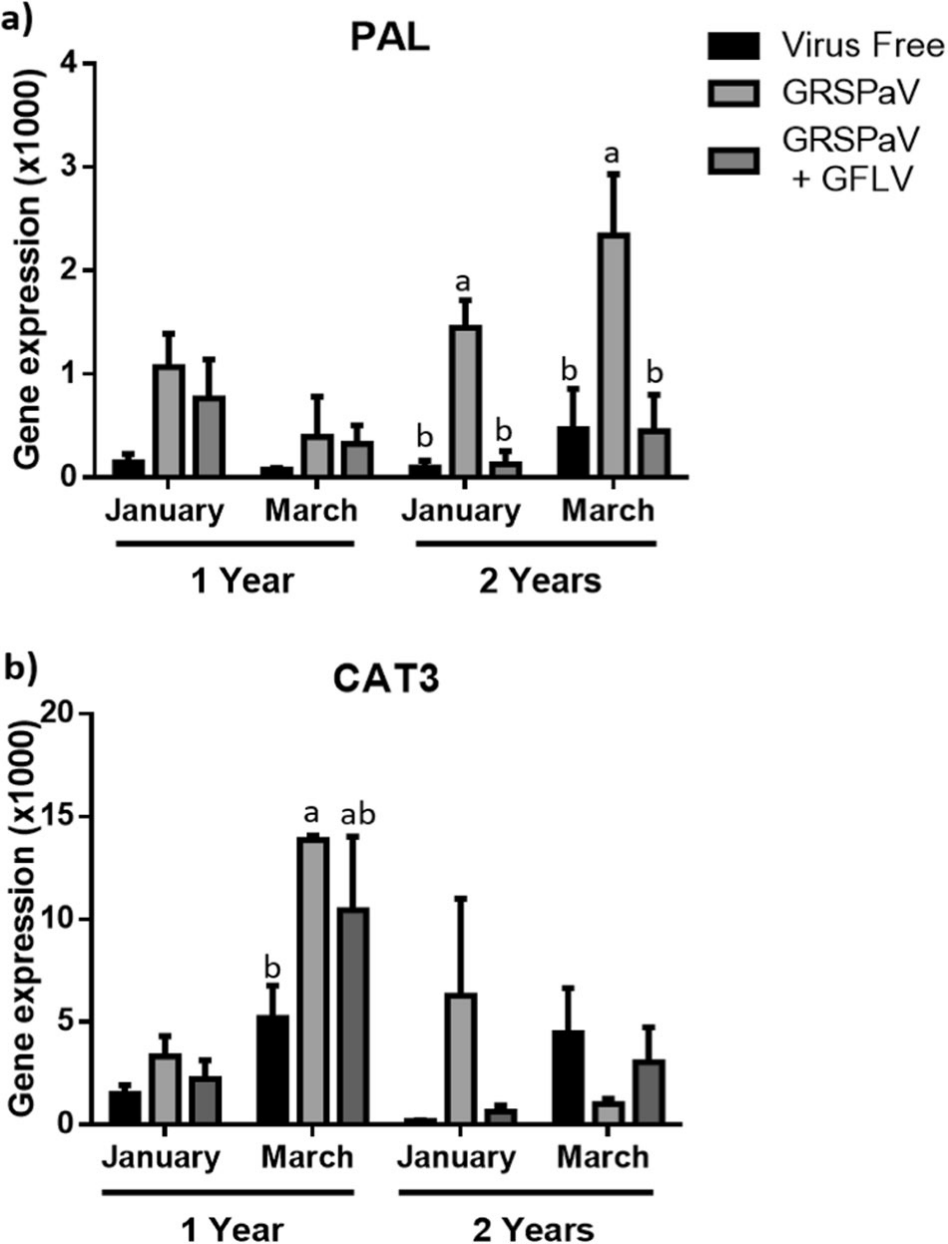
Relative expression of genes involved in secondary metabolism. The relative expression of *PAL* (**a**) and *CAT3* (**b**) was evaluated. The graphed expression levels were evaluated in January (BBCH 75) and March (BBCH 89) of 2018 in one- and two-year-old plants and are presented relative to the reference gene, ubiquitin, and include three biological replicates and two technical repetitions. The data were analysed by one-way ANOVA to compare between phytosanitary conditions. The graphs show the mean ± SE. Different letters indicate significant differences.

Finally, the *PAL* and *CAT3* gene expression was measured, and both genes were involved in the secondary metabolism of the grapevine (Fig. 8). The *PAL* expression was greater in two-year-old plants with simple GRSPaV infections in both January and March (Fig. 8a). A similar expression profile was observed in one-year-old plants, but these differences were not significant (Supplementary Table 12). Regarding the *CAT3* levels, one-year-old plants infected with GRSPaV showed higher expression of this gene in March (Supplementary Table 13), and higher average *CAT3* expression was observed in samples collected in January from one- and two-year-old plants (Fig. 8b). No differences in the *CAT3* expression were observed for any of the other phytosanitary statuses.

## Discussion

The characterisation of viral variants present in plants used in this work shows a high nucleotide identity of isolate CAS 61 with the viral variant GRSPaV-SY, described for the first time in plants with declining Syrah symptoms in California, United States^16^. In contrast, a previous work that characterised Chilean isolates of GRSPaV showed a high nucleotide identity of these samples with the groups of the GRSPaV-SG and GRSPaV-1 variants^17^. Regarding GFLV, this same work showed that the majority of Chilean GFLV isolates converge into two groups of viral variants: GFLV Ch1 and Ch2. However, one of the Chilean isolates, ChFL785 had high sequence similarity with US isolates. The results obtained here show a high nucleotide identity of the CAS-AV with sequences belonging to the ChFL785 isolate and the US WAPN57 isolate obtained from grapevines cultivar Pinot Noir^18^.

The growth and photosynthetic parameters were measured in one- and two-year-old plants with different phytosanitary statuses. When the growth parameters were studied in the one-year-old plants (Figs. 3 and 4), no differences were observed between the groups under different phytosanitary conditions, whereas in the two-year-old plants, greater vigour was observed in GRSPaV-infected grapevines, which was explained by their greater number of leaves per shoot as well as the greater elongation of the main shoot, a product of a greater number and length of internodes. These results differ from others previously reported for different cultivars, in which GRSPaV was found to have minimal or no effect on the vigour of “Albano”^8^, “Madeleine Sylvaner”, “Ortega”^9^ and “Savagnin rose”^10^. However, all those studies measured the vigour as the average pruning weight and were mostly performed under field conditions in five- to eight-year-old plants, so these and other experimental differences make it difficult to establish a comparison. Moreover, Pantaleo and collaborators^12^ detected that GRSPaV infected plants exhibited a lower expression of miR156 and a higher miR172 expression, a distinctive miRNA pattern expression for the transition of juvenile to adult vegetative phase^19–24^. GRSPaV also reduced the expression of miR171, which would have an important role in the regulation of vegetative growth and reproductive organs development^25–27^ via modulation of gibberellin and auxin homeostasis^28^. Thus, it could be possible that the higher plant growth of GRSPaV infected plants observed in the present work could be related to an early entry into the adult vegetative phase.

Regarding the growth results in plants infected with GRSPaV and GFLV, this group of plants did not show significant differences in relation to virus-free plants. These results are unexpected since there are important negative effects from the GFLV virus on the plant vigour^7^ and total shoot length^29^. However, in those papers, no GRSPaV detection analyses were reported, and the authors did not mention anything about a possible co-infection with GRSPaV, a virus that is almost impossible to detect without molecular analyses; this makes it difficult to establish a comparison with the results shown here for plants with mixed infections. Furthermore, reduced plant growth is frequently associated with the development of typical fanleaf disease symptoms. By contrast, the results presented here were evaluated in asymptomatic plants that did not present fanleaf disease, since the ones that began to show symptoms developed accelerated decay and death, therefore we could not incorporate their data into the results (data not shown).

Regarding the photosynthesis measurements, the *P*_n_ levels in one-year-old plants did not show significant differences between the different phytosanitary conditions, although plants with simple GRSPaV infections tend to exhibit higher values for this parameter. By contrast, it is possible to appreciate a tendency towards reduced *P*_n_ values in two-year-old plants as their phytosanitary status worsens, this reduction becomes statistically significant in two-year-old plants with double infections. The relatively low *P*_n_ values obtained in this study probably occurred because the measurements were performed on plants growing in greenhouses. The *P*_n_ values are concordant with the C_i_ results obtained here, since the plants that showed higher *P*_n_ levels have significantly lower *C*_i_ values. This trend in the behaviour of photosynthetic parameters is similar to the one observed by Gambino et al.^11^.

Finally, no significant differences were observed in the *g*_s_ and *E*, independent of the phytosanitary status, which confirms the previous evidence that the photosynthetic reduction caused by viral infections is not a consequence of stomatal closure in leaves^11,30^.

It is interesting to note that in the case of photosynthetic measurements, plants with mixed viral infections presented a more severe alteration with respect to the simple GRSPaV-infected and virus-free plants, contrary to what was observed in the growth measurements in Figs. 3 and 4. Therefore, the presence of GRSPaV and GFLV viral infections in one- and two-year-old plants would reduce the ability to fix CO_2_ without significantly reducing the shoot elongation during the early stages of *V. vinifera* development. However, with these results, it would be expected that the vigour of the co-infected plants would be affected during the stages of grapevine development to follow as reported in the literature.

In general, despite the limited number of replications (between 3 and 8) utilised in this study to quantify changes in growth and physiological variables, it was possible to report clear tendencies and statistically significant differences among the groups of different sanitary condition. This was due to the low variability within each group, represented by the standard errors (SE), which was likely a reflection of the strong consequence of the virus on the affected variables.

Regarding the effect of the viral infections on the expression levels of some selected genes, the first studied genes were some of the primary regulators of the GA pathway, a plant hormonal route associated with plant growth and elongation^31,32^. It was possible to observe that for *DELLA1*, a basal inhibitor of the GA response pathway, although the mean gene expression is higher in GRSPaV-infected plants, the variability of the data did not allow for the establishment of significant differences between the three analysed groups. Regarding repressors of *DELLA1*, the basal overexpression of *GID1b* and *SLY1a* could indicate the possibility of a higher *DELLA1* degradation rate, which could influence the sensitivity of the plant to GA and would explain, at least partially, the overexpression of GA response genes, such as *GASA1, 3* and *6*, in plants infected with GRSPaV. It is interesting to note that at the end of the summer, two-year-old plants infected with GRSPaV consistently exhibited a basal expression of the three evaluated genes. The lower expression level of these genes could involve a reduction in the GRSPaV effect due to, for instance, reduced virus replication or another issue that we have not considered in this study. Additional studies are necessary for a better comprehension of these results.

Moreover, previous works showed that GRSPaV reduce the levels of miR167 in infected plants^12^. MiR167 have been described to negatively regulate several genes associated with stress and development^33^. ARF6 and ARF8 are two target genes of miR167, both genes are necessary for the right floral development in several plants, such as *Arabidopsis thaliana*^34^, tomato^35^ and soybean^36^. In addition, it has been reported that DELLA avoid the union of a protein complex constituted by ARF6, BZR1 and PIF4, which in the absence of DELLA, join the promotor region of genes related to cellular elongation of several tissues^37^. Therefore, the higher expression levels of SLY1a and GID1b detected here (Fig. 6b, c), and the deregulation of miR167 by GRSPaV reported in the literature, could be complementary mechanisms by which the virus promotes the conformation of the ARF6/BZR1/PIF4 complex and induce the expression of GA-response genes (like GA1, 3 and 6) (Fig. 6), increasing plant growth (Figs. 3 and 4).

Plants with double viral infections did not show differences in the expression levels of GA response genes in comparison with virus-free plants, which is consistent with the plant growth results described above. Therefore, it is possible that part of the differences observed in the evaluated growth parameters of plants with simple GRSPaV infections may be explained by the increased levels of GA response genes. Effects that would be cancelled when the GRSPaV infection is accompanied by GFLV infection.

The changes in the expression of genes related to photosynthetic processes (*LHCII* and *ACD1*) were consistent with those reported by Gambino and collaborators^11^, which would support the basal overexpression of these genes in GRSPaV-infected plants. Additionally, the overexpression of *LHCII* and *ACD1* produced by GRSPaV is lost when there is a co-infection with GFLV, which could explain the detrimental effect of the double infection on the measured photosynthetic parameters. It is interesting to observe that the average expression of *ACD1* in double-infected plants is higher than its expression in virus-free plants. This finding may have occurred because the GFLV replicative mechanism leads to greater chlorophyll degradation, which could explain the increased *ACD1* expression in comparison to the virus-free plants.

Finally, the quantification of *PAL*, a precursor for the phenylpropanoid pathway, and therefore a variety of secondary metabolites associated with defence processes, growth and plant development^38^, showed an expression profile similar to the aforementioned genes, in which GRSPaV-infected plants exhibited higher levels of gene expression than the other groups of plants. Although it is interesting to highlight that the greatest increase in *PAL* expression was observed in two-year-old plants infected with GRSPaV, unlike most of the previous results, in which increased gene expression was registered in one-year-old plants. Increased *PAL* expression has also been observed in response to other viral infections such as the GLRaV-3 infection^39^ as well as other pathogens, such as *Plasmopora viticola*^40^, or the nematode *Xiphinema index*^41^, which is a vector of GFLV. By contrast, the expression of *CAT3*, the gene associated with cell detoxification induced by stress, is higher in one-year-old plants. Double-infected plants showed higher average *CAT3* expression than virus-free plants, in most cases and consistent with other reports, which is also congruent with the results obtained for the *ACD1* gene, another gene related to cell detoxification.

In summary, the results submitted here allow us to provide new evidence about the effect of GRSPaV on one- and two-year-old *V. vinifera* plants. Specifically, our findings indicate that the presence of GRSPaV would have a positive effect on different growth parameters and shoot elongation caused in conjunction with the increased activation of genes involved in the GA response pathway, despite showing a lower net photosynthetic rate and lower CO_2_ assimilation. Plants with simple GRSPaV infections also exhibited an overexpression of genes related to photosynthetic and cell detoxification processes. The integration of these results to previous works that showed a reduced defence response in GRSPaV infected plants^11^ suggest that the virus might interfere gibberellin regulation to modify the defence-growth crosstalk^13^.

In addition, the double infection with GRSPaV and GFLV did not affect the shoot elongation during early stages of plant growth, although the damage caused to the gas exchange parameters at this stage of grapevine development could modify these results during future seasons and could lead to the development of the typical symptoms caused by GFLV.

These results confirm the co-evolution hypothesis between GRSPaV and *V. vinifera* proposed by Gambino^11^, and suggest a positive confirmation of a beneficial effect from GRSPaV on the early developmental stages of grapevines “Cabernet Sauvignon”, in accordance with the symbiotic mutualism theory proposed by Pantaleo^12^.

From an agronomic point of view, these data confirm the scarce possibility of visually evaluating the presence of GRSPaV and especially GRSPaV and GFLV in mixed infections, in plants acquired by growers for vineyard establishment. It is only through the application of a sampling protocol and the performance of laboratory tests that it is possible to ascertain the health status of the plant material.

## Materials and methods

Evaluations of the growth and physiological parameters and the quantification of the expression levels of genes involved in the processes of interest were performed in plants with three different phytosanitary conditions.

### Plant material

*V. vinifera* plants from the “Cabernet Sauvignon” cultivar, clone BKN B R2.0011, were analysed by qPCR detection for the 8 most important viruses, namely GLRaV-1^42^, -2^43^ and -3^44^; GVA and GVB^45^, GFLV^46^, GRSPaV^47^ and GFkV^48^. Plants with three different phytosanitary statuses were selected: virus-free, GRSPaV-infected and GRSPaV- and GFLV-infected plants.

The selected grapevines were multiplied in vitro and then acclimated during the spring of 2016 (two-year-old plants) and 2017 (one-year-old plants). After that, the plants were established in 3-L pots in greenhouses located at the San Joaquín Campus of Pontificia Universidad Católica de Chile (Santiago, Chile) under controlled temperatures (21 °C + 3 °C) and humidity (RH: 60% ± 10%) conditions. The pots were arranged in a completely random design consisting of three to eight biological replicates per phytosanitary status per plant age.

### GRSPaV and GFLV molecular characterisation

RNA was extracted from leaves for the GRSPaV virus and phloem for the GFLV virus, using PureLink Minikit (Thermo Fisher) and the TRIS reagent (Sigma-Aldrich), respectively. For synthesis of first-strand cDNA Affinity Super Script (Agilent) was used. Viral cDNA was PCR amplified in 20 µL reaction containing 1× Mg-free buffer, 2.5 mM MgCl_2_, 5 pmol of each primer RSP-35F and RSP-36R^3^ for GRSPaV and CP1F (5′-GAGCCCAGACTGAGCTCAAC-3′), CP2R (5′-AGTCCATAGTGGTCCCGTTC-3′), CP3F (5′-ACATTTGTGCGCCAATCTTC-3′) and CP4R (5′-CGCCACTAAAAGCATGAAAC-3′)^17^ for CP of RNA2 of GFLV, 0.2 mM dNTP mix, 1 unit of taq DNA platinum polymerase (Thermo Fisher) and 5 µL of the reverse transcription mixture. Thermal cycling conditions were one cycle at 94 °C for 4 min; 35 cycles at 94 °C for 30 s, 55 °C for 30 s and 72 °C for 30 s; and a final extension at 72 °C for 7 min for GRSPaV and one cycle at 94 °C for 4 min; 35 cycles at 94 °C for 30 s, 52 °C and for 30 s and 72 °C for 1 min; and a final extensión at 72 °C for 10 min for GFLV.

### Phylogenetic analysis

Multiple sequence alignments of RdRP of GRSPaV (Supplementary Table 1) and RNA 2 of GFLV (Supplementary Tables 2 and 3), and nucleotide identity levels were performed with GeneiousPrime. The phylogenetic analysis was performed using the Neighbour-Joining (NJ) and Maximun Likehood methods in the MEGA X analysis package. A bootstraps value for each node of NJ tree was calculated using 1000 and 500 replicates for GRSPaV and GFLV, respectively.

### Growth measurements

The monitoring of the growth of plants under different phytosanitary conditions was performed by evaluating the following parameters: the shoot elongation, number and length of the internodes and the number of leaves per shoot. To facilitate a comparison between replicates, the plants were managed such that the growth of a single main shoot was allowed, and the growth of lateral shoots was prevented.

Growth measurements were performed in the one-year-old plants using 6 to 8 biological replicates per phytosanitary status. For the two-year-old plants, the measurements included 3 to 8 replicates per group.

The evaluations were performed between October (BBCH 12) 2017 and March (BBCH 89) 2018 for the two-year-old plants and between November (BBCH 15) 2017 and March (BBCH 89) 2018 for the one-year-old grapevines. During this time, the parameters mentioned above were measured every 4 weeks.

### Evaluating gas exchange parameters

During January of 2018, the following parameters were evaluated: the photosynthetic net rate (*P*_n_), leaf internal CO_2_ concentration (*C*_i_), stomatal conductance (*g*_s_) and leaf transpiration rate (*E*). For these measurements, an infrared gas analyser (IRGA) Handheld Photosynthesis System model CI-340 (CID BIO-Science) was used.

For optimised measurement, the plants were removed from the greenhouses and evaluated outdoors between 10 a.m. and 1 p.m., and the measurements were performed using mature leaves. Between 3 and 7 biological replicates were used for each phytosanitary condition. The resulting data were analysed using analysis of variance (ANOVA).

### RNA extraction and quantification of relative expression levels

The leaf samples were collected in January and March of 2018 from three different groups of plants. These months correspond to grapevine growth stages BBCH 75 and BBCH 89, respectively^49^. The samples were frozen in liquid nitrogen and stored at −80 °C.

RNA was extracted using a 3% CTAB protocol modified from Yu and collaborators^50^. The quantity and quality of the extracted RNA were determined using both fluorometer (Qubit 4, Thermo Fisher Scientific) and Nanodrop (Nanodrop 2000, Thermo Fisher Scientific) instruments. The cDNA synthesis was performed with an Affinity Script QPCR cDNA synthesis kit (Agilent Technologies) according the manufacturer´s instructions, beginning with 0.5 µg of RNA. Real-time PCR was performed using 2 µL of cDNA and Brilliant II SYBR Green QPCR Master mix (Agilent Technologies), with an Mx3000P detection system (Stratagene). The primer sequences used here were obtained from the literature as follows: *ACT*^51^, *UBQ*^52^, *DELLA1, DELLA3, GID1b* and *SLY1a*^53^, *GASA1, GASA3* and *GASA6*^54^, *LHCII, ACD1, PAL* and *CAT*^11^ and *GAPDH*^55^. The qPCR conditions were: 95 °C for 10 min for initial denaturation, followed by 40 cycles of 95 °C for 30 s, 60 °C for 30 s and 72 °C for 30 s and a final extension at 95°C for 1 min, 55 °C for 30 s and 95 °C for 30 s. The gene expression levels were normalised using the reference genes ubiquitin (*UBQ)* and actin (*ACT)*. The relative expression of the evaluated genes was calculated using the comparative *C*_t_ method (2^−ΔΔCt^)^56^ with three biological replicates and two technical replicates. The specificity of reaction was monitored by evaluating the dissociative curves at the end of every qPCR. The gene expression was calculated and graphed as the mean and standard deviation.

### Identification and quantification of viruses

In order to identify of GRSPaV strains present in the plant material, four samples corresponding to the clone 61 (internal code of the clone used here) were used to amplify a fragment of RdRp of GRSPaV. The primers and PCR conditions were obtained from the literature^57^. The PCR product was cloned into pGEMT-easy vector system (Promega, Madison, Wisconsin, USA) following the manufacter’s instructions. Plasmid was purified using the plasmid miniprep kit (Qiagen, Valencia, CA, USA) and sequenced. These sequences were compared with sequences obtained from each of the GenBank complete genome sequences and included in the analysis. Nucleotide sequence corresponding region of Apple stem pitting virus (ASPV, accession number D21829) was retrieved from GenBank and used as an outgroup. Phylogenetic analysis was performed using both the Neighbour Joining and the Maximum Likelihood methods.

Additionally, the concentrations of GRSPaV and GFLV were quantified in the same samples. The primer, Taqman sequences and detection protocol used here were obtained from the literature^58^. In order to obtain a higher complementarity with Chilean GFLV isolates, an additional Taqman probe (GFLV CP2-Chp)^17^ was included in the GFLV quantification protocol, whose sequence is 5′-TTAGTGAGTGGAACGGGACCACTATGGA-3′.

### Statistical analyses

The results for the growth monitoring, physiological parameters and gene expression levels collected from plants of the same ages and different phytosanitary statuses were analysed by one-way ANOVA. Additionally, multifactorial comparisons of the gene expression levels were performed between plants with different ages and equal phytosanitary statuses by two-way ANOVA. In both cases, a Fisher's mean comparison was performed and the significant differences between the means were assigned considering values of *p* < 0.005.

## Supplementary information

Supplementary Material.
