# Supplementary material for: Divergent molecular and growth responses of young “Cabernet Sauvignon” (Vitis vinifera) plants to simple and mixed infections with Grapevine rupestris stem pitting-associated virus
Source: Hortic Res. 2020 Jan 1;7:2. doi: 10.1038/s41438-019-0224-5 (PMC6938478; doi:10.1038/s41438-019-0224-5)
Supplement: Supplementary file 1 — Supplementary Material. [file 41438_2019_224_MOESM1_ESM.pdf]

<u>Supplementary Figures</u>

Viral load quantification

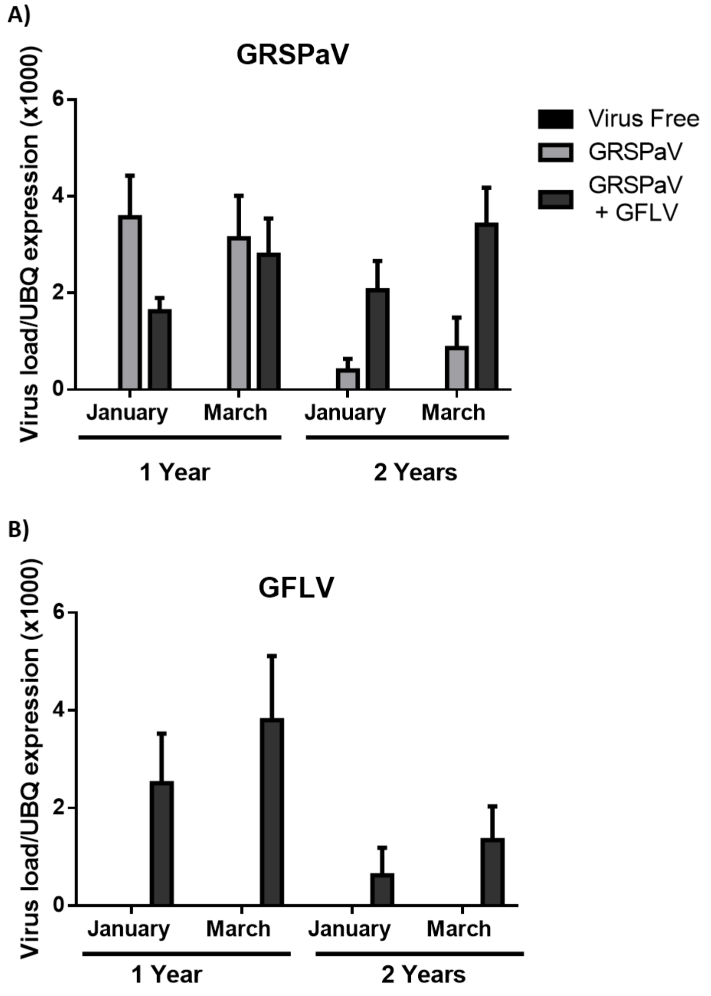

Supplementary Figure 1: Quantification of viral load. The viral concentration quantifications of GRSPaV (A) and GFLV (B) were performed in January and March in samples of virus-free, infected with GRSPaV and infected with GRSPaV and GFLV plants from 1- and 2-year-old grapevines. A two-way ANOVA was performed to compare the viral load levels between phytosanitary statuses and plant ages. The graphs show the mean <u>+</u> SE.

<u>Supplementary Tables</u>

Supplementary Table 1. Information of isolates utilized in phylogenetic analysis of GRSPaV

Isolate	Host	Accession Number
GRSPaV-GG	*V. riparia* cv. Grande Glabre	JQ922417
GRSPaV-SG1	*V. rupestris* cv. St George	AY881626
GRSPaV-BS	Hybrid Bertille Seyve 5563	AY881627
GRSPaV-SY	*V. vinifera* cv.Syrah	AY368590
GRSPaV-PN	*V vinífera* cv. Pinot noir	AY368172
GRSPaV-PG	*V vinífera* cv. Pinot Gris	HE591388
GRSPaV-MG	*V vinífera* cv. Moscato Giallo	FR691076
GRSPaV-WA	*V vinífera* cv. Merlot/Cabernet Franc	KC427107
GRSPaV-3138-07	*V. vinifera* cv. Unknown	JX559646
GRSPaV-JF	*V. labruscana* cv. Kyoho	KR054734
GRSPaV-LSL	*V. vinifera* cv. Riesling	KR054735
GRSPaV-VF	*V. flexuosa*	KT948710

Supplementary Table 2. Chilean Isolates utilized in phylogenetic analysis of GFLV.

Name isolate	Host	Geographic Region of Chile	Accession Number
Ch1	Cabemet Sauvignon	Metropolitana	KC256955
Ch70	Chardonnay	Valparaíso	KC256964
Ch81	Cabernet Sauvignon	Metropolitana	KC256966
Ch92	Cabernet Sauvignon	Metropolitana	KC256962
Ch162	Muscat	Coquimbo	KC256957
Ch177	Red Globe	Coquimbo	KC256958
Ch785	Cabernet Sauvignon	Metropolitana	KC256965
Ch1100	Cabernet Sauvignon	Metropolitana	KC256954
Ch4217	Superior	Atacama	KC256956
Ch4406	Thompson Seedless	Atacama	KC256959
Ch4532	Superior	Atacama	KC256960
Ch4598	Superior	Atacama	KC256961

Supplementary Table 3. Information of isolates utilized in phylogenetic analysis of GFLV

Isolate	Host	Accession Number
A17a	*V vinífera* cv. Chardonnay	AY370956
A17b	*V vinífera* cv. Chardonnay	AY780900
A17d	*V vinífera* cv. Chardonnay	AY780901
F13	*V vinífera* cv. Muscat	X16907
Vol5lc3	*V vinífera* cv. Volovnik	DQ922664
Vol55c3	*V vinífera* cv. Volovnik	DQ922673
NW	*V vinífera* cv. Huxel	AY017338
Ghu	*V vinífera* cv. Gloria Hungariae	AY371026
WACF2142	*V vinífera* cv. Cabemet Franc	GQ332371
WACH911	*V vinífera* cv. Chardonnay	GQ332364
WAME1492	*V vinífera* cv. Merlot	GQ332370
WAPN57	*V vinífera* cv. Pinot noir	GQ332367
WAPN6132	*V vinífera* cv. Pinot noir	GQ332366
CACSBl	*V vinífera* cv. Cabemet Sauvignon	GU972576
CACSB5	*V vinífera* cv. Cabemet Sauvignon	GU972580
CACSC3	*V vinífera* cv. Cabemet Sauvignon	GU972583
CAZINA1	*V vinífera* cv. Zinfandel	GU972571
CAZINA2	*V vinífera* cv. Zinfandel	GU972572
Gdef	Ch.amaranticolor	AY291208
NWArMV	*V vinífera* cv. Pinot Gris	AY017339

Supplementary Table 4: Effect of the phytosanitary status and plant age on the *DELLA1* expression.

**DELLA1 expression**			
		**Month of sampling**	
		**January**	**March**
**Age of plant**	**(expression levels)**		
1 year		0.000077 **ns**	0.000044 **ns**
2 year		0.000025	0.000003
**ANOVA**	***df***	***P*- value**	
Age of Plant	1	0.092	0.353
Phytosanitary Status	2	0.528	0.437
Age of Plant x Phytosanitary status	2	0.785	0.388
Residual	12		

Means followed by different letters indicate significant differences, while those marked with the initials “ns” indicate that there were no significant differences, considering $p < 0,05$ and using Fisher's comparison test.

Supplementary Table 5: Effect of the phytosanitary status and plant age on *GID1b* expression.

**GID1b expression**			
		**Month of sampling**	
		**January**	**March**
**Age of plant**	**(expression levels)**		
1 year		0.283 **ns**	2.587 **B**
2 year		0.188	1.501 **A**
**ANOVA**	***df***	***P*- value**	
Age of Plant	1	0.286	0.037
Phytosanitary Status	2	0.27	0.058
Age of Plant x Phytosanitary status	2	0.809	0.107
Residual	12		

Means followed by different letters indicate significant differences, while those marked with the initials “ns” indicate that there were no significant differences, considering p < 0,05 and using a Fisher comparison test.

Supplementary Table 6: Effect of the phytosanitary status and plant age on *SLY1a* expression.

**SLY1a expression**			
		**Month of sampling**	
		**January**	**March**
**Age of plant**	**(expression levels)**		
1 year		0.00255 **ns**	0.00369 **B**
2 year		0.00174	0.00122 **A**
**ANOVA**	***df***	***P*- value**	
Age of Plant	1	0.18	<0.001
Phytosanitary Status	2	0.043	0.008
Age of Plant x Phytosanitary status	2	0.655	0.011
Residual	12		

Means followed with different letters indicate significant differences, while those marked with the initials “ns” indicate that there were no significant differences, when considering $p < 0,05$ and using a Fisher comparison test.

Supplementary Table 7: Effect of the phytosanitary status and plant age on *GASA1* expression.

GASA1 expression			
		**Month of sampling**	
		**January**	**March**
**Age of plant**	**(expression levels)**		
1 year		0.00608 **A**	0.00788 **A**
2 year		0.01340 **B**	0.01820 **B**
**ANOVA**	***df***	***P*- value**	
Age of Plant	1	0.008	0.036
Phytosanitary Status	2	<0.001	0.387
Age of Plant x Phytosanitary status	2	0.051	0.414
Residual	12		

Means followed by different letters indicate significant differences, while those marked with the initials "ns" showed no significant differences, considering p < 0,05 and using a Fisher comparison test.

Supplementary Table 8: Effect of the phytosanitary status and plant age on *GASA3* expression.

**GASA3 expression**			
		**Month of sampling**	
		**January**	**March**
**Age of plant**	**(expression levels)**		
1 year		0.02010 **ns**	0.000267 **B**
2 year		0.00021	0.000006 **A**
**ANOVA**	***df***	***P*- value**	
Age of Plant	1	0.34	0.002
Phytosanitary Status	2	0.4	0.008
Age of Plant x Phytosanitary status	2	0.396	0.012
Residual	12		

Means followed by different letters indicate significant differences, while those marked with the initials “ns” had no significant differences, considering p < 0,05 and using a Fisher comparison test.

Supplementary Table 9: Effect of the phytosanitary status and plant age on *GASA6* expression.

GASA6 expression			
		**Month of sampling**	
		**January**	**March**
**Age of plant**	**(expression levels)**		
1 year		0.1214 **A**	0.2224 **B**
2 year		0.1767 **B**	0.0928 **A**
**ANOVA**	***df***	***P*- value**	
Age of Plant	1	0.018	<0.001
Phytosanitary Status	2	0.003	0.015
Age of Plant x Phytosanitary status	2	0.621	0.026
Residual	12		

Means followed by different letters indicate significant differences, while those marked with the initials “ns” indicate that there were no significant differences, considering $p < 0,05$ and using a Fisher comparison test.

Supplementary Table 10: Effect of the phytosanitary status and plant age on *LHCII* expression.

**LHCII expression**			
		**Month of sampling**	
		**January**	**March**
**Age of plant**	**(expression levels)**		
1 year		0.3690 **B**	0.0711 **ns**
2 year		0.1960 **A**	0.0517
**ANOVA**	***df***	***P*- value**	
Age of Plant	1	0.018	0.207
Phytosanitary Status	2	0.012	0.052
Age of Plant x Phytosanitary status	2	0.848	0.195
Residual	12		

Means followed by different letters indicate significant differences, while those marked with the initials "ns" indicate that there were no significant differences, considering p < 0,05 and using a Fisher comparison test.

Supplementary Table 11: Effect of the phytosanitary status and plant age on *ACD1* expression.

**ACD1 expression**			
		**Month of sampling**	
		**January**	**March**
**Age of plant**	**(expression levels)**		
1 year		0.0132 **B**	0.0116 **B**
2 year		0.0020 **A**	0.0059 **A**
**ANOVA**	***df***	***P*- value**	
Age of Plant	1	<0.001	0.003
Phytosanitary Status	2	0.004	0.093
Age of Plant x Phytosanitary status	2	0.01	0.313
Residual	12		

Means followed by different letters indicate significant differences, while those marked with the initials “ns” showed no significant differences, considering p < 0,05 and using a Fisher comparison test.

Supplementary Table 12: Effect of the phytosanitary status and plant age on *PAL* expression.

**PAL expression**			
		**Month of sampling**	
		**January**	**March**
**Age of plant**	**(expression levels)**		
1 year		0.00066 **ns**	0.00027 **A**
2 year		0.00056	0.00116 **B**
**ANOVA**	***df***	***P*- value**	
Age of Plant	1	0.605	0.01
Phytosanitary Status	2	0.001	0.024
Age of Plant x Phytosanitary status	2	0.146	0.075
Residual	12		

Means followed by different letters indicate significant differences, while those marked with the initials "ns" showed no significant differences, considering p < 0,05 and using a Fisher comparison test.

Supplementary Table 13: Effect of the phytosanitary status and plant age on *CAT3* expression.

**CAT3 expression**			
		**Month of sampling**	
		**January**	**March**
**Age of plant**	**(expression levels)**		
1 year		0.00235 **ns**	0.00983 **B**
2 year		0.00237	0.00284 **A**
**ANOVA**	***df***	***P*- value**	
Age of Plant	1	0.992	<0.001
Phytosanitary Status	2	0.152	0.417
Age of Plant x Phytosanitary status	2	0.476	0.03
Residual	12		

Means followed with different letters indicate significant differences, while those marked with the initials “ns” showed no significant differences, considering p < 0,05 and using a Fisher comparison test.
